# Apelin–APLNR pathway across development, inflammation, and vascular remodeling: an endothelial perspective

**DOI:** 10.1038/s12276-026-01760-w

**Published:** 2026-07-02

**Authors:** Hongryeol Park, Ralf H. Adams, Kee-Pyo Kim

**Affiliations:** 1 Max Planck Institute for Molecular Biomedicine, Department of Tissue Morphogenesis, Münster, Germany; 2 Department of Medical Life Sciences, College of Medicine, The Catholic University of Korea, Seoul, Republic of Korea

**Keywords:** Angiogenesis, Development

## Abstract

The apelinergic system, composed of the ligands Apelin and Elabela and their shared receptor APLNR, has key functions in vascular development and endothelial homeostasis. Originally identified as an orphan G protein-coupled receptor, APLNR is now recognized as a receptor highly enriched in endothelial cells across organs, where it integrates developmental cues, mechanical forces, hypoxia, and inflammatory signals. Apelin and Elabela guide angiogenic patterning during cardiac, pulmonary, and retinal development, and in adulthood they support endothelial nitric oxide production, vascular stability, and tissue adaptation to stress. Pathological conditions expose the dependence of endothelial integrity on this pathway. Reduced Apelin availability or altered *APLNR* expression contributes to hypertension, cardiac remodeling, pulmonary vascular injury, and immune-driven inflammation. Conversely, restoring apelinergic signaling improves endothelial barrier function, limits leukocyte recruitment, and mitigates fibrotic and ischemic damage, including in experimental models of autoimmune encephalomyelitis and acute lung injury. Therapeutic interest in this pathway has increased with the development of stabilized Apelin analogs and small-molecule APLNR agonists that have longer half-lives than native peptides. Together, emerging evidence positions the Apelin–APLNR axis as a context-dependent regulator of endothelial function and a promising therapeutic target in cardiovascular, pulmonary, and inflammatory diseases.

## Apelin–APLNR axis enhances endothelial function and stability

The Apelin receptor (APLNR) has a crucial role in endothelial signaling, integrating vascular, metabolic, and inflammatory signals. This Review highlights the Apelin–APLNR axis’s role in endothelial cells (ECs), focussing on expression dynamics, regulatory mechanisms, and organ-specific functions. It integrates evidence from single-cell RNA sequencing, in vitro studies, and in vivo models to explore how APLNR expression and its ligands, Apelin and Elabela, shape EC function and vascular homeostasis. Apelin signaling has been shown to promote vasodilation, support endothelial barrier integrity, and reduce inflammation through ECs. This Review further suggests that targeting the Apelin–APLNR pathway may benefit cardiovascular and inflammatory diseases, but clinical translation will require careful attention to context-dependent signaling, ligand bias, peptide stability, and patient stratification.

This summary was initially drafted using artificial intelligence, then revised and fact-checked by the author.

## Introduction

The Apelin receptor (APLNR, also known as APJ) has emerged as a key endothelial signaling regulator that integrates vascular, metabolic, and inflammatory cues. APLNR is a G protein-coupled receptor initially identified based on its sequence similarity to the angiotensin II type 1 receptor (AT1R), a member of the rhodopsin family^1^. Before its endogenous ligands were discovered, APJ was considered an orphan receptor^2^. The first ligand, Apelin, was isolated from bovine stomach extracts^2^, followed by the discovery of Elabela, encoded by the *Apela* gene (also referred to as Toddler in zebrafish), which has a crucial role in embryogenesis, particularly in cardiac progenitor migration and endoderm differentiation^3–5^. Unlike Apelin, which is widely expressed postnatally across multiple tissues, APELA expression is strongest during early embryonic stages, where it regulates cell migration and early developmental processes in zebrafish^3,5^. In mammals, APELA is additionally expressed in extraembryonic structures such as the placenta, where it contributes to cardiovascular development and maternal–fetal homeostasis^6^.

Apelin, encoded by *Apln*, named by Masahiko Fujino’s group, was initially found to be expressed in multiple rat tissues, including the mammary gland, lung, spinal cord, brain, heart, and white adipose tissue. Among its isoforms, Apelin-13 and Apelin-36 were particularly abundant in the mammary gland and lungs^7^. More recent transcriptomic analyses, including the Mouse Cell Atlas, confirmed *Apln* expression in the ovary, heart, omentum, adrenal gland, and muscle^8^.

APLNR has a similarly broad distribution. In mice, it is expressed in the heart, adrenal gland, uterus, lung, placenta, and skin^8^, whereas in humans, the Human Protein Atlas reports its expression in the brain, stomach, liver, testis, heart, skeletal muscle, and lung. According to the Cancer Genome Atlas, APLNR is also expressed in various primary tumors, including those of the brain, lung, colon, breast, and adrenal gland^9^. Despite this widespread distribution, recent single-cell RNA-sequencing studies have demonstrated that *APLNR* expression is predominantly localized to endothelial cells (ECs)^10^, underscoring its vascular specificity under homeostatic conditions. This endothelial-specific expression supports a prominent role for apelinergic signaling in regulating endothelial function and vascular homeostasis.

ECs form the inner lining of blood vessels and act as the interface between the bloodstream and surrounding tissues. They regulate vascular tone, permeability, leukocyte trafficking, and diverse organ-specific vascular functions^11^. Single-cell analyses have shown that capillary ECs exhibit extensive molecular heterogeneity across tissues, reflecting their specialized roles^12^. For example, in the lung, *Car4*⁺ ECs facilitate gas exchange, whereas gCap ECs mediate immune cell trafficking^13^. In the kidney, glomerular ECs are fenestrated to support filtration, whereas peritubular capillaries mediate solute reabsorption and secretion^14^. Endothelial dysfunction in these beds leads to organ-specific pathology: disruption of the glomerular filtration barrier causes proteinuria and chronic kidney disease^15^; peritubular injury promotes hypoxia and fibrosis^16^; pulmonary endothelial impairment contributes to gas exchange failure and pulmonary hypertension^17^; and breakdown of the blood–brain barrier allows neurotoxic infiltration in neurodegenerative diseases such as Alzheimer disease^18^. In this context, Apelin levels are reduced in multiple pathological conditions. Circulating Apelin levels is decreased in multiple cancer types^19^, heart failure^20^, and obstructive hypertrophic cardiomyopathy^21^, as well as in mouse models of experimental autoimmune encephalomyelitis (EAE)^19^. Administration of Apelin-13 (A13) alleviates disease symptoms in several contexts, highlighting its therapeutic potential in restoring endothelial function.

**Fig. 1:**
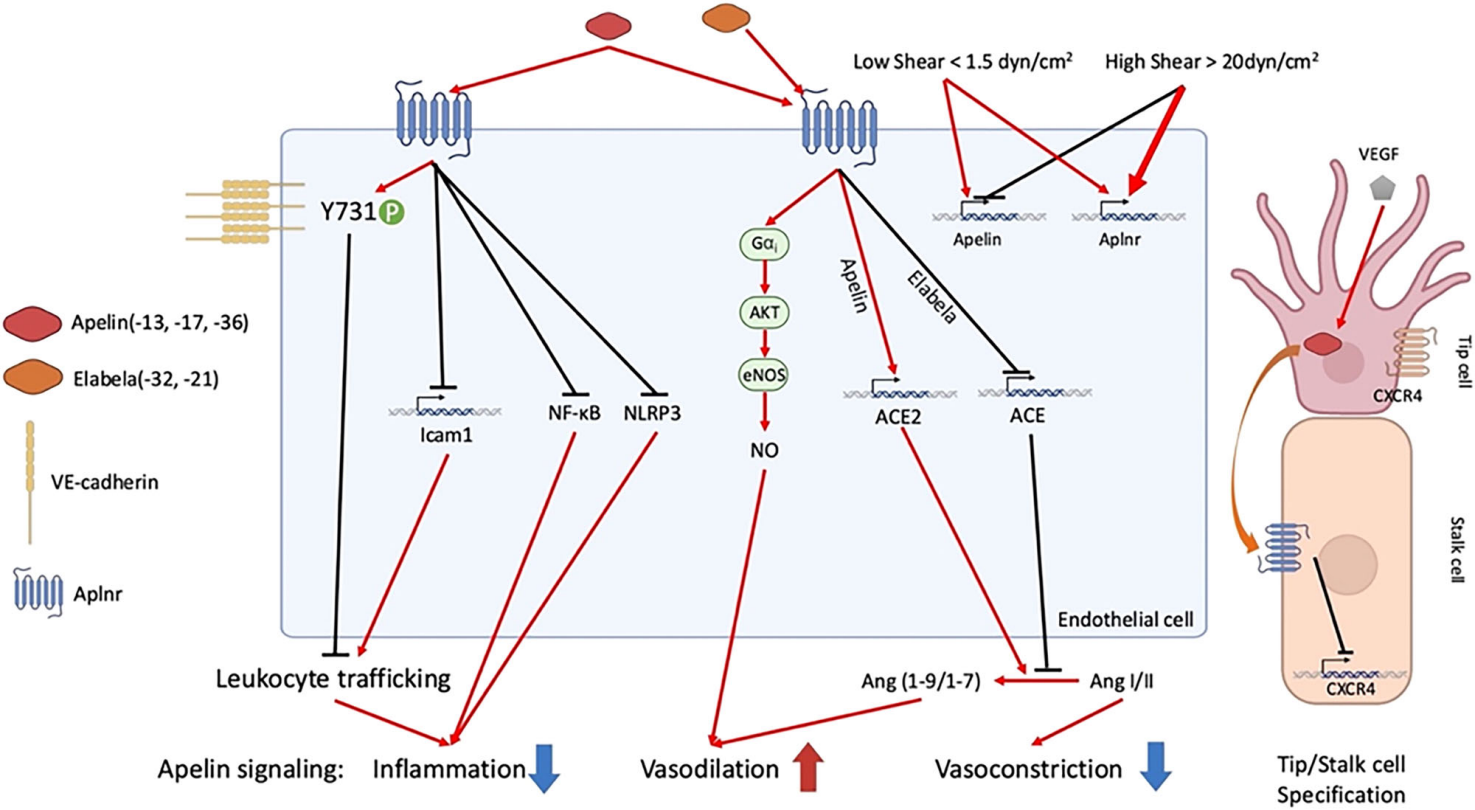
Endothelial Apelin–APLNR signaling programs. Apelin peptides (Apelin-13, Apelin-17, and Apelin-36) and Elabela bind to APLNR expressed on endothelial cells. APLNR activity is dynamically regulated by environmental cues, including shear stress (low versus high flow), hypoxia, and interactions with vascular endothelial growth factor (VEGF) and CXCR4 signaling, which together shape endothelial activation states and angiogenic responses. In the context of angiogenic sprouting, these signals contribute to endothelial cell specification, influencing tip versus stalk cell behavior through coordination with VEGF–CXCR4 pathways. Upon receptor activation by ligand, APLNR primarily engages G protein-dependent signaling, leading to PI3K-Akt-mediated endothelial nitric oxide synthase (eNOS) phosphorylation and nitric oxide (NO) production, thereby promoting vasodilation. In parallel, APLNR signaling stabilizes endothelial junctions via vascular endothelial (VE)-cadherin and suppresses pro-inflammatory pathways (for example, intercellular adhesion molecule 1 (ICAM1), nuclear factor (NF)-κB, NLR family pyrin domain containing 3 (NLRP3)), resulting in reduced leukocyte trafficking and vascular inflammation. Apelin–APLNR signaling further interfaces with the ACE2 axis to counterbalance Ang II-mediated vasoconstriction, contributing to the regulation of vascular tone. Collectively, these pathways converge to regulate key endothelial functions, including angiogenic patterning, vascular remodeling, barrier integrity, inflammation, and vasomotor control. The signaling output is context-dependent and exhibits ligand bias, with G protein-mediated pathways predominantly associated with vascular protection. ACE, angiotensin-converting enzyme.

Together, these findings support an endothelial-centric model of Apelin-APLNR signaling, in which receptor expression and ligand availability dynamically shape vascular responses across physiological and pathological contexts. Figure 1 summarizes the major endothelial signaling programs through which Apelin–APLNR regulates angiogenic patterning, vasomotor control, barrier integrity, inflammatory activation, and vascular remodeling. In the following sections, we highlight emerging insights into the Apelin–APLNR axis in ECs, focussing on the expression dynamics, regulatory mechanisms, and organ-specific functions of this pathway.

## Apelin, Elabela, and APLNR: expression and processing

### Apelin expression and its regulation in ECs

Apelin, an endogenous ligand of APLNR, is expressed across multiple tissues. Single-cell RNA-sequencing data, including results from the Mouse Cell Atlas, show that within mice, Apelin transcripts are most highly enriched in ECs and astrocytes^8^. Although Apelin was initially categorized as an adipokine based on bulk tissue analysis and in vitro adipocyte models^22^, recent single-cell RNA-sequencing studies have shown that *Apln* transcripts are scarcely detectable in mature adipocytes in vivo^23^. This discrepancy likely reflects the extensive use of 3T3-L1 cells, which originate from mouse embryonic fibroblasts and are commonly differentiated into adipocyte-like cells in vitro^24–27^. Even studies using directly isolated adipocytes have primarily quantified Apln expression by qPCR, which measures relative transcript changes under culture conditions rather than absolute in vivo expression levels^28^.

Aldosterone (Ald), a mineralocorticoid hormone secreted by the adrenal cortex, is a well-known regulator of blood pressure^27^. In vivo studies have shown that Ald treatment reduces Apln mRNA levels in adipose tissue and lowers circulating Apelin peptide concentrations. Consistent with this, patients with essential hypertension exhibit significantly reduced plasma Apelin levels^29^. These findings suggest that Ald may negatively regulate *Apln* expression in peripheral tissues. Although *Apln* transcripts are largely absent in mature adipocytes in vivo^23^, Ald suppresses *Apln* transcription in cultured adipocytes^27^. The mineralocorticoid receptor (MR) is also expressed in ECs, including those in adipose tissue, vasculature, and the heart. Because MR activation in ECs induces proinflammatory gene expression, reduces nitric oxide (NO) availability, and promotes oxidative stress, Ald–MR signaling may similarly repress *Apln* expression in ECs. However, direct evidence for Ald-mediated transcriptional repression of *Apln* in ECs is presently lacking. The potential interplay between Ald–MR signaling and endothelial Apelin regulation will be discussed in a later section in the context of vascular homeostasis and pathological remodeling.

*Apln* expression in ECs has been well documented across several developing organs, even before the advent of single-cell transcriptomics. In situ hybridization and reporter assays have demonstrated *Apln* expression in the endothelium of the retina^30^, as well as in the heart, lungs, and quadriceps muscle^31^. More recent single-cell analyses have expanded this expression profile to include stromal cell types such as fibroblasts and astrocytes.

The transcriptional regulation of *Apln* in ECs is mediated in part by hypoxia. A functional hypoxia responsive element located in the first intron of the human *APLN* gene (+813 to +826) is directly activated by hypoxia-inducible factor 1 alpha (HIF-1α)^32^. Binding sites for additional transcription factors, including activator protein 1, USF, NF1, and AP4, are present in the core promoter region, although the mechanisms by which these factors regulate *Apln* expression in ECs remain to be defined^33^.

Apelin has a key role in angiogenesis and its expression is particularly prominent during retinal vascular development in mice and intersegmental vessel formation in zebrafish^34,35^. In situ hybridization studies have shown that *Apln* is enriched in endothelial tip cells, the leading cells at the forefront of vascular sprouting, rather than in stalk cells that experience shear stress from blood flow^36^. This spatially restricted expression is consistent with the functional role of Apelin in tip cell behavior.

Several growth factors and signaling pathways regulate *Apln* expression in ECs. Vascular endothelial growth factor (VEGF) induces *Apln* transcription, as shown in synovial membrane cells^37^, and in vivo VEGF administration increases the number of *Apln*-positive ECs in skeletal and cardiac muscle^38^, supporting its role as a positive regulator of Apelin expression. NOTCH signaling also modulates Apelin levels. In human umbilical vein endothelial cells (HUVECs), inhibition of NOTCH signaling upregulates *APLN* expression, whereas stimulation with Delta-like ligand (DLL)4-coated surfaces, which activate NOTCH, suppresses it^35^. Because NOTCH suppresses VEGFR2 expression, the regulation of *APLN* by NOTCH signaling may be partly mediated by altered VEGF responsiveness. Mechanical stimuli such as shear stress further influence *APLN* expression. In vitro studies using HUVECs have shown that low shear stress (1.5 dyn/cm^2^) enhances *APLN* transcription, whereas high shear stress (20 dyn/cm^2^) suppresses it^39^. This may help explain the relatively low *Apln* expression observed in stalk cells, which are directly exposed to blood flow, in contrast to tip cells.

### Peptide processing and bioactivity regulation of Apelin

Apelin is synthesized as a 77-amino-acid prepropeptide that undergoes sequential proteolytic processing to generate its active isoforms. Furin, a calcium-dependent serine endoprotease localized in the trans-Golgi network, cleaves proapelin (55 amino acids) into bioactive fragments of 36, 17, 13, and 12 amino acids, all of which are capable of binding to APLNR and initiating downstream signaling cascades^40^. Following intracellular cleavage, Apelin peptides are further modified by extracellular endopeptidases, influencing their stability and bioavailability. Enzymes such as kallikrein and ACE2 cleave the N-terminal and C-terminal regions of Apelin, leading to partial inactivation and increased susceptibility to degradation. Notably, neprilysin (NEP) cleaves Apelin at a critical site between Arg and Leu site required for APLNR binding, completely abolishing its biological activity^41–43^. As a result, circulating Apelin peptide levels may not accurately reflect Apelin functional activity. Although NEP is expressed in multiple tissues including the kidney and intestine, its regulatory effects on Apelin have been best characterized in vascular and plasma compartments^44–46^. Among endogenous isoforms, [Pyr^1^]Apelin-13 is considered the most potent because its N-terminal pyroglutamate modification, generated by glutaminyl peptide cyclotransferase, enhances both its stability and the affinity of the isoform for APLNR. Consequently, [Pyr¹]Apelin-13 is widely used in experimental models to study EC signaling^47^.

### Elabela expression and its transcriptional regulation

Elabela was first identified as a non-coding transcript but was later recognized as a secreted ligand of the APLNR^3,48^. During embryogenesis, *Apela* is expressed from the onset of zygotic genome activation and becomes enriched in mesendodermal progenitors undergoing gastrulation movements^48^. At later stages, its expression localizes to the midline mesoderm, where it guides angioblast migration^49^. In mammals, *Apela* is expressed in embryonic stem cells and extraembryonic tissues such as the placenta, where it has crucial roles in cardiovascular development and maternal fetal homeostasis^4,6,50^. Transcriptional regulation of *Apela* is tightly linked to developmental signaling networks. In embryonic stem cells, *Apela* is highly expressed under pluripotent conditions^3,51^, and its transcription is repressed by p53 activation in mouse embryonic stem cells^52^. Beyond embryogenesis, *Apela* expression has also been reported in adult tissues, including the kidney, prostate, and endothelium^3,51^, where it may contribute to vascular homeostasis. Under pathological conditions, *Apela* transcription is responsive to hypoxia and retinoic acid, and its expression is increased in several tumor types, particularly ovarian and kidney clear cell carcinoma^53^. *Apela* is further upregulated in the placenta under maternal stress conditions^6^, suggesting that its transcriptional regulation extends beyond developmental contexts into stress and disease environments.

### Peptide processing and bioactivity of Elabela

Elabela is translated as a 54-amino-acid preproprotein that contains a predicted N-terminal signal peptide, which may facilitate its secretion^3,54^. Proteolytic processing of this precursor generates several isoforms such as ELA-32, ELA-21, and ELA-11, with differing potency and plasma stability^54,55^. Structure–activity relationship studies identified key C-terminal residues in ELA-32, including His26, Arg28, Val29, Pro30, and Phe31, as essential for APLNR binding and receptor activation. These features distinguish Elabela from Apelin, whose bioactivity is largely determined by its N-terminal residues^55^. Biochemical profiling has shown that ELA-32 and ELA-21 have nanomolar affinity for APLNR and effectively suppress cAMP production, with potencies comparable to [Pyr^1^]Apelin-13. Both peptides also show stronger β-arrestin recruitment than [Pyr^1^]Apelin-13, which indicates a bias toward β-arrestin-mediated signaling. ELA-11 exhibits lower overall receptor affinity, but its effects on cAMP suppression and β-arrestin recruitment remain similar to those of [Pyr^1^]Apelin-13 (ref. ^56^). Post-translational modifications add an additional layer of regulation. For example, the N-terminal pyroglutamate form of ELA-32 ([Pyr^1^]ELA-32) has increased metabolic stability, prolonging its presence in plasma in vitro^54^. Elabela contains two conserved cysteine residues, but the physiological significance of these cysteine residues remains under investigation^57^.

### Transcriptional regulation of APLNR

Early insights into *Aplnr* gene regulation came from rodent studies. In rats, the 5ʹ flanking region of the *Aplnr* gene contains binding sites of several transcription factors, including those for specificity protein 1, the glucocorticoid receptor, estrogen receptor α, CCAAT enhancer-binding protein, and activator protein 1. Among these elements, a specificity protein 1 binding site located between −585 bp and −686 bp was identified as essential for promoter activity^58^. In humans, more direct evidence for promoter regulation has been demonstrated in nasopharyngeal carcinoma (NPC). Treatment of NPC cell lines with all-*trans*-retinoic acid markedly enhanced *APLNR* promoter activity. Luciferase reporter assays in NPC cell lines (5–8F and HNE1) showed that the promoter region spanning −1,765 bp to −820 bp is particularly responsive to all-*trans*-retinoic-acid stimulation^59^, indicating potential disease-specific and context-dependent regulatory mechanisms.

Although these promoter studies were performed primarily in non-EC types, regulation of *APLNR* in ECs is more closely tied to environmental cues. Krüppel-like factor 2 (KLF2), a transcription factor responsive to shear stress, is a central regulator in this setting. *Aplnr* is among several flow responsive genes, and its expression is significantly reduced following KLF2 knockdown in ECs, including those isolated from the lung^36,39,60,61^. Upregulation of *APLNR* mRNA in HUVECs under both low shear stress (1.5 dyn/cm^2^) and high shear stress (20 dyn/cm^2^) further supports the idea that *APLNR* transcription is sensitive to a broad range of mechanical forces^39^. Hypoxia is another strong inducer of *Aplnr* transcription. Multiple studies have shown that hypoxic conditions elevate *Aplnr* expression in diverse tissues, mediated in part by HIF-1α^62^. In glioblastoma, tumor-associated ECs express markedly higher levels of both Apelin and APLNR, along with increased VEGFA expression, compared with normal brain ECs^19,63^. These findings support a model in which hypoxia induces VEGF production, and VEGF promotes Apelin expression, although additional validation is needed to define the exact regulatory cascade.

Apelin may also enhance *Aplnr* expression, indicating the presence of a positive feedback loop in this signaling axis. Under flow conditions, Apelin treatment increases *APLNR* mRNA levels in HUVECs^60^. Conversely, *Apln* knockout models show reduced Aplnr expression in intestinal tissues^64^. Such feedback may amplify endothelial sensitivity during angiogenic or pathological conditions. One proposed model suggests that hypoxia-induced VEGF expression elevates *Apln* levels in endothelial tip cells. Apelin may then act in a paracrine manner to induce *Aplnr* expression in neighboring stalk cells. Spatial analyses show that *Aplnr* is largely absent in VEGF-responsive tip cells but is expressed in stalk cells located behind the leading edge of the VEGF gradient. This pattern suggests that APLNR induction is not directly triggered by VEGF but may instead be driven indirectly through HIF-1α-mediated Apelin expression, adding an additional layer to its spatial and temporal regulation^62^.

## Endothelial Apelin–APLNR signaling and downstream programs

### Proximal receptor signaling and endothelial effects

Upon ligand binding, APLNR activates Gα_i/o_ and Gα_q/11_ proteins, which in turn activate downstream signaling pathways such as the PI3K-Akt, ERK1 and ERK2, and AMPK pathways in ECs^65^. Among Apelin isoforms, Apelin-13 induces Akt phosphorylation and receptor desensitization more efficiently than Apelin-36 in HUVECs, although both peptides stimulate ERK1 and ERK/2 activation to a similar degree^66^. A key vascular response to Apelin signaling is the Akt-mediated phosphorylation of endothelial NO synthase (eNOS), which enhances NO production and promotes vasodilation^67,68^. Apelin-17 further increases Akt and eNOS phosphorylation in ECs and simultaneously reduces tumor necrosis factor α (TNFα)-induced monocyte adhesion. This anti-inflammatory effect is abolished by the eNOS inhibitor L-NAME, confirming its dependence on NO^67^. APLNR also forms a functional heterodimer with the bradykinin B2 receptor, and this interaction cooperatively enhances eNOS activation and NO release, illustrating how G protein-coupled receptor crosstalk contributes to vascular signaling complexity^68,69^.

Recent structural studies, including cryogenic electron microscopy analyses of APLNR–Gi complexes, have provided mechanistic insights into how APLNR generates context-dependent signaling outputs^70^. These studies demonstrate that distinct ligands stabilize specific receptor conformations associated with divergent signaling profiles. Peptide ligands such as Apelin and synthetic agonists, including MM07 and CMF-019, engage residues within the orthosteric binding pocket in distinct ways, leading to differential conformational rearrangements within the receptor core^70,71^.

Structural analyses further identified two critical interaction regions (“twin hotspots”) that interact with the C-terminal portion of Apelin peptides and contribute to signaling bias^70^. These ligand-specific interactions propagate through an allosteric network connecting the ligand-binding pocket to the intracellular transducer-binding interface, thereby modulating the balance between G protein activation and β-arrestin recruitment. Together, these findings indicate that APLNR signaling output is not solely determined by receptor activation per se, but by ligand-specific conformational states that bias downstream signaling responses.

Beyond classical G protein-mediated signaling, APLNR can activate β-arrestin-dependent pathways, particularly under mechanical stimulation. In HEK293 cells, mechanical stretch increases β-arrestin recruitment and ERK activation independently of Apelin or G protein activity^72^. Whether similar mechanisms operate in ECs remains unclear, especially in the lungs where *Aplnr* is expressed in gCap endothelium that experiences continuous mechanical forces during respiration^13^. Such mechanosensitivity may be relevant in vascular beds subject to repetitive stretch. APLNR activation also mobilizes intracellular calcium through Gα_q/11_. This may influence endothelial contraction, cytoskeletal dynamics, and vascular permeability, although these roles have not yet been fully defined in vivo.

Apelin signaling further contributes to endothelial barrier function by regulating cell–cell junctions. Inflammatory or hypoxic conditions disrupt tight and adherens junction proteins such as VE-cadherin and ZO-1. Treatment with Apelin-13 restores VE-cadherin localization and reduces transendothelial immune cell migration, underscoring its role in maintaining vascular integrity^60^. In addition, Apelin reinforces transcriptional programs essential for endothelial homeostasis. It increases the expression of KLF2, a transcription factor responsive to shear stress. These regulatory circuits support endothelial quiescence, in part by promoting KLF2-dependent transcription of eNOS and subsequent NO production^61,73^. Together, these interactions highlight how the Apelin–APLNR axis stabilizes endothelial function under both physiological and pathological conditions. Recent zebrafish studies have expanded this framework by showing that tissue-derived Apelin directs venous-specific angiogenesis in a Notch-independent manner^74^. In this context, Aplnra and Kdrl cooperate within a subset of venous ECs to drive VEGFA-induced sprouting toward the neural tube. These cells, termed L-tip cells, are distinct from Dll4-Notch-regulated arterial tip cells and are exposed to VEGFA and Apelin originating from the neural parenchyma. Apelin signaling stabilizes these nascent sprouts by promoting pericyte recruitment and maintaining perfusion, underscoring its role in developmental angiogenesis. Taken together, these findings position the Apelin–APLNR axis as a central regulator of endothelial signaling and vascular stability, with downstream outcomes that extend into transcriptional and epigenetic remodeling.

### Gene-regulatory mechanisms in ECs

Beyond its immediate signaling effects, Apelin also modulates endothelial gene expression. In HUVECs, Apelin-13 induces cytoplasmic translocation of the histone deacetylases (HDAC4) and HDAC5 through Gα_13_-mediated phosphorylation, thereby altering transcriptional activity^61^. By contrast, APJ knockout embryos display nuclear accumulation of these HDACs in cardiac ECs, indicating that Apelin–APLNR signaling is required for proper epigenetic regulation during vascular development. A key downstream transcription factor is KLF2, which is essential for vascular homeostasis. Absence of Apelin reduces *KLF2* expression, whereas APLNR overexpression activates the KLF2 promoter, likely through autocrine Apelin secretion from ECs^61,73^. Because KLF2 upregulates both Apelin and APLNR, this creates a positive feedback loop that reinforces sustained Apelin signaling in ECs^36,39,60^.

In human pulmonary artery ECs, APLN knockdown suppresses FGF2 and FGFR1 expression through upregulation of the miR-424-503 axis^75^. Conversely, under hypoxic conditions, downregulation of miR-503 in bone-marrow-derived endothelial progenitor cells increases APLN expression, revealing a negative feedback mechanism that modulates Apelin signaling during hypoxia^76^. Another microRNA, miR-139-5p, which is induced by flow and targets CXCR4, is also influenced by Apelin–APLNR activity. APLNR knockdown reduces miR-139-5p levels and consequently elevates CXCR4 expression^36,75^. These relationships illustrate how Apelin shapes transcriptional and post-transcriptional regulatory networks.

## Apelin, Apela, and Aplnr signaling in vascular development of the heart, lungs, and retina

### Cardiac development

During early embryogenesis, Elabela–Aplnr signaling is indispensable for cardiac morphogenesis. In zebrafish, deficiency of either *aplnr* or *apela* results in early cardiac defects and embryonic lethality, whereas *apln* mutants show milder or delayed cardiac abnormalities, consistent with a later functional requirement^3,48,50,77^. These developmental defects arise from impaired migration of cardiac and angioblast progenitors, a process regulated in part by the Nodal and transforming growth factor-beta (TGFβ) pathways^5,49^. Temporal profiling in zebrafish demonstrates that Elabela is expressed during early gastrulation, preceding angioblast formation and acting upstream in cardiovascular development^48,49^. Apelin participates in vascular development as well, but its later onset suggests a distinct temporal and functional role^48^.

In mice, loss of *Aplnr* similarly disrupts early cardiovascular development. Although detailed embryonic phenotyping of global *Aplnr* knockout models is limited, existing studies report early embryonic lethality or markedly reduced survival, often accompanied by cardiovascular and placental defects^4,50,73^. By contrast, *Apln* knockout mice are viable and fertile but develop progressive cardiac dysfunction in adulthood. Their impaired hemodynamic responses during pressure overload or aging highlight a postnatal role for Apelin in maintaining cardiovascular homeostasis^77^. Together, these findings support a temporal division of labor in which Elabela–Aplnr signaling is required for early cardiac morphogenesis, whereas Apelin–Aplnr signaling becomes essential for later cardiovascular adaptation and stress resilience.

Beyond progenitor migration, the Elabela–Aplnr axis is also required for coronary vasculature formation. In mice, deletion of either *Apela* or *Aplnr* disrupts coronary vessels derived from the sinus venosus and impairs plexus expansion. Nevertheless, compensation by endocardium-derived progenitors preserves overall cardiac function^78^. These findings reinforce a model in which Elabela–Aplnr signaling is crucial for early morphogenesis and coronary patterning, whereas Apelin–Aplnr signaling functions mainly in cardiovascular adaptation under physiological stress^77^.

### Lung development

Apelin–Aplnr signaling functions are expressed early during lung formation. In rats, both proteins are detectable from embryonic day 13.5, and their expression gradually declines toward adulthood^79^. Notably, although monomeric and oligomeric forms of Aplnr decrease in adults, the dimeric form increases^79^. Biochemical studies further show that inhibition of Aplnr dimerization alters the Apelin-induced G protein binding profile^80^, suggesting that developmental changes in receptor assembly may influence Apelin signaling strength and quality, although this has yet to be validated in vivo.

Functional evidence for Apelin signaling during lung morphogenesis comes from explant culture studies. Treatment of embryonic lung explants with [Pyr^1^]Apelin-13 reduces overall growth, decreases phosphorylation of p38 and JNK1 and JNK2, and increases ERK1 and ERK2 phosphorylation at high concentrations (10^−^^5^ M)^79^. Although APLNR expression was initially reported in both epithelial and endothelial layers, the epithelial signal likely resulted from limited histological resolution, as these layers lie in close proximity at distal branching sites. Subsequent single-cell RNA-sequencing studies demonstrated that *Aplnr* expression is restricted to ECs, indicating that MAPK signaling changes originate primarily from endothelial activation. Endothelial ERK1 and ERK2 activity promotes extracellular matrix remodeling and microvascular stabilization^81^, supporting the idea that Apelin signaling fine-tunes endothelial organization rather than driving epithelial branching morphogenesis.

Single-cell RNA sequencing of fetal mouse and human lungs reveals that *Aplnr* expression is enriched in ECs, particularly in general capillary (gCap) cells that regulate vasomotor tone and immune interactions^13,82^. By contrast, *Apln* transcripts are concentrated in *Car4*⁺ aerocytes, the endothelial subtype specialized for gas exchange^13,82^. This complementary distribution suggests a paracrine circuit in which Apelin produced by *Car4*⁺ aerocytes activates neighboring *Aplnr*⁺ gCap cells to coordinate endothelial maturation and formation of the alveolar capillary interface. Although still hypothetical, this model provides a plausible mechanism for intraendothelial communication during alveolar vascular development. Because global *Aplnr* knockout mice die around embryonic day 10.5 from heart failure^73^, direct assessment of *Aplnr* function in lung morphogenesis is limited. However, lineage tracing using *Aplnr*-CreERT2 and related single-cell analyses have confirmed the endothelial-restricted expression pattern described earlier^13,60^. Although endothelial-specific deletion of *Aplnr* does not impair embryonic vascular formation^83^, adult mutants exhibit pulmonary vascular pruning and elevated right ventricular systolic pressure, indicating that Apelin signaling, although dispensable for early morphogenesis, is required for maintaining endothelial integrity and alveolar capillary homeostasis after birth. Together, these findings indicate that Apelin–Aplnr signaling functions primarily within ECs during lung development. Through modulation of ERK-dependent endothelial activity, receptor dimerization dynamics, and coordination between gCap and *Car4*⁺ aerocytes endothelial populations, the pathway contributes to the maturation and long-term stability of the pulmonary microvasculature.

### Retinal development

During postnatal retinal angiogenesis, *Apln* and its receptor *Aplnr* are strongly upregulated at the sprouting vascular front. Endothelial tip cells are enriched for *Apln*, whereas adjacent stalk or venous side ECs show higher Aplnr expression^36,84,85^. This complementary distribution supports a short-range paracrine circuit that stabilizes tip and stalk organization. In neonatal mice, intraperitoneal administration of Apelin peptide increases vessel area and tip cell number. Although the mature superficial vasculature at postnatal day 10 is unchanged, deeper layer formation is enhanced, indicating that Apelin primarily functions during the early angiogenic phase^86^. Loss-of-function studies further delineate this developmental window. *Apln* knockout mice exhibit a transient delay in both superficial and deep retinal vascularization between postnatal days 5 and 15, yet the vasculature normalizes by adulthood, suggesting compensatory mechanisms or overlapping ligand activity^85^. By contrast, endothelial-specific *Aplnr* deletion causes sustained defects in vessel density and branching, accompanied by dysregulated CXCR4 signaling in ECs^36^.

At the molecular level, VEGF induces *Apln* expression in tip cells, whereas Notch restricts *Apln* expression in neighboring ECs. This interplay reinforces spatially confined angiogenic behavior and maintains tip and stalk polarity^30,85^. Hemodynamic cues also influence *Apln* and *Aplnr* transcript levels, with different shear stress levels modulating their expression and NO signaling. This provides an additional mechanism for fine-tuning endothelial behavior once blood flow is established^39^. Two additional observations explain why *Apln* increases apparent tip cell activity despite *Aplnr* being enriched in stalk or venous ECs. First, pharmacological studies and oxygen-induced retinopathy models show that Apelin released from the vascular front acts on Aplnr in adjacent ECs, regulating their proliferation and spatial arrangement. Blocking Aplnr reduces pathological tufting and disorganized endothelial expansion^87^. Second, Apelin signaling induces a pro-angiogenic metabolic state by increasing c-Myc and glycolysis, enabling sustained sprout extension even when receptor expression is relatively higher in nearby stalk cells^35^.

The role of Elabela in normal retinal development remains unclear because its transcripts are scarce under physiological conditions. Nevertheless, Elabela supplementation reduces vaso-obliteration and vascular leakage in oxygen-induced retinopathy, suggesting a protective function during vascular stress^88^. The eventual normalization of *Apln* knockout mouse retinas further implies the presence of compensatory mechanisms, potentially involving Elabela signaling or flow-dependent APLNR activation. Together, these observations suggest that overlapping ligand activity and hemodynamic cues contribute to maintaining a balanced and functional vascular network in the postnatal retina.

## Apelin–APJ signaling in cardiovascular disease

### Blood pressure regulation via endothelial APLNR–NO axis

Apelin is a key regulator of vascular homeostasis and blood pressure, acting primarily through endothelial APLNR activation and NO production^89^. Ligand binding to APLNR activates the PI3K-Akt pathway, leading to phosphorylation of eNOS at Ser1177 and subsequent NO synthesis that promotes vasodilation^90,91^. Although ERK1 and ERK2 may also be engaged, PI3K-Akt remains the dominant mechanism for eNOS activation across vascular beds.

Immunocytochemical studies have detected APJ protein in both vascular smooth muscle and endothelial layers in human vessels, and cultured vascular smooth muscle cells respond directly to Apelin by displaying altered differentiation and growth behavior^92,93^. Human vascular studies further confirm an endothelial requirement for Apelin-mediated vasodilation. In human mammary arteries, Apelin-13, Apelin-36, and [Pyr¹]Apelin-13 induce endothelium-dependent relaxation through cyclooxygenase-derived mechanisms, and this response is abolished by endothelial denudation^47^. Overall, these findings indicate that Apelin primarily targets ECs, rather than smooth muscle cells, in humans.

Disease models underscore the physiological importance of endothelial Apelin–APLNR signaling. Circulating Apelin levels are reduced in patients with hypertension and in rodent models of heart failure, both of which involve endothelial dysfunction^29,94,95^. *Aplnr* knockout mice exhibit normal baseline blood pressure but fail to respond to Apelin administration^96^, demonstrating that the pathway is not required for resting vascular tone but is essential for stimulus-induced, NO-dependent vasodilation. In diabetic mice, Apelin-induced vasorelaxation is blocked by eNOS inhibition, confirming that the hypotensive effects of Apelin depend on endothelial NO synthesis^91^. Beyond peripheral circulation, central APLNR in circumventricular sensory organs modulates the depressor response to peripherally administered Apelin, suggesting that central APLNR contributes to blood pressure regulation under hypertensive conditions^97^. Receptor-proximal signaling bias further modulates APLNR function in the vascular system. Distinct ligands can differentially engage G protein-dependent versus β-arrestin-mediated pathways, resulting in divergent vascular responses^70^. In particular, G protein-biased APLNR agonists preferentially enhance PI3K-Akt–eNOS signaling and NO-mediated vasodilation, whereas increased β-arrestin recruitment may limit these effects or promote receptor desensitization^98^. Consistent with this, the small-molecule agonist CMF-019 exhibits a strong bias toward G protein signaling with minimal β-arrestin recruitment and demonstrates improved vasodilatory efficacy compared with endogenous Apelin peptides^71^. These findings highlight the therapeutic potential of selectively targeting G protein-biased APLNR signaling to optimize cardiovascular outcomes.

### RAAS crosstalk and heart failure progression

Apelin signaling interacts directly with the renin–angiotensin–aldosterone system (RAAS), adding a hormonal regulatory component to its vascular functions. A key point of intersection is ACE2, an enzyme that converts Angiotensin (Ang) II into Ang-(1–7), a peptide with vasodilatory and anti-inflammatory properties^99,100^. Apelin increases ACE2 expression in endothelial and cardiac tissues^101^, and genetic studies in mice have shown that loss of Apelin reduces ACE2 levels in failing hearts. Conversely, pharmacological or genetic inhibition of AT1R, or treatment with Ang-(1–7), mitigates cardiac hypertrophy and dysfunction^101^. In vitro analyses further demonstrate that Apelin enhances *ACE2* promoter activity, supporting a direct positive influence of Apelin–APLNR signaling on the ACE2–Ang-(1–7) axis in failing myocardium^101^. Interestingly, ACE2 also cleaves Apelin peptides. ACE2-derived Apelin fragments show reduced APLNR signaling capacity, including diminished Akt and eNOS phosphorylation, compared with full-length peptides^42^. Thus, ACE2 both promotes Apelin-driven Ang-(1–7) formation and simultaneously limits Apelin bioactivity by peptide processing. This dual behavior establishes a reciprocal regulatory loop in which Apelin enhances ACE2–Ang-(1–7) signaling, whereas ACE2 constrains the duration and magnitude of Apelin signaling.

Changes in Apelin–APLNR expression accompany heart failure progression. Myocardial APLNR density is decreased in failing human hearts^102^, and circulating Apelin levels decline in parallel with worsening systolic function^103^. These reductions likely impair responsiveness to endogenous Apelin during advanced disease stages and weaken its ability to counteract RAAS activation.

Elabela also interacts with RAAS pathways. The Elabela–APJ axis protects against pressure overload-induced heart failure and Ang II-mediated cardiac injury by reducing hypertrophy and dysfunction in experimental models, in part through downregulation of ACE, which contrasts with Apelin-mediated ACE2 upregulation^104^. In the kidney, Elabela suppresses the intrarenal renin–angiotensin system, lowers blood pressure, and limits renal damage by attenuating RAAS activation^105^. In high salt-loaded spontaneously hypertensive rats, Elabela is more effective than Apelin-13 in reducing blood pressure, cardiorenal dysfunction, fibrosis, and hypertrophy. These benefits are linked to counter-regulation of the ACE, ACE2, and NEP arms of the RAAS in the heart and kidney^106^. Together, these findings position the Apelin–ACE2 and Elabela–RAAS circuits as essential regulators of vascular tone and heart failure progression.

### Re-evaluating cell-type-specific APLNR functions in cardiac remodeling

Pressure overload models revealed that APLNR function is highly context-dependent across cardiac cell types. Endothelial-specific Aplnr deletion results in severe left ventricular failure following transverse aortic constriction, with reduced fractional shortening and maladaptive wall remodeling, despite preserved baseline systolic function^104,107^. These findings indicate that endothelial APLNR is essential for maintaining microvascular support of cardiac function during chronic hemodynamic stress.

Cardiomyocyte-specific Aplnr deletion has been reported to produce a contrasting phenotype, with relative preservation of fractional shortening after transverse aortic constriction compared with controls^107^. A mechanosensory role for APLNR has been proposed in cardiomyocytes, in which mechanical stretch induces hypertrophic signaling, whereas Apelin binding shifts signaling toward G protein-coupled pathways that oppose hypertrophy while preserving contractile function^72,107^. However, these findings should be interpreted with caution in light of more recent single-cell transcriptomic and lineage-tracing studies showing that APLNR expression in the adult heart is predominantly restricted to endothelial and perivascular populations, with only low expression in cardiomyocytes^60,108^. Thus, proposed cardiomyocyte-autonomous functions of APLNR may reflect developmental, context-dependent, or indirect paracrine mechanisms rather than a dominant receptor program in adult cardiomyocytes.

Consistent with this interpretation, global loss-of-function studies reveal non-equivalent phenotypes between ligand and receptor deficiency. Global Apelin deficiency leads to age-dependent declines in contractility and exacerbated systolic dysfunction following pressure overload^77^, indicating that endogenous Apelin is required for adaptive remodeling. By contrast, global APLNR deletion abolishes both Apelin-mediated protective signaling and ligand-independent stretch transduction, resulting in a net protective effect against the transition from hypertrophy to heart failure^72^. These divergent phenotypes highlight the dual nature of APLNR as both a ligand-dependent receptor and a mechanosensitive signaling node, while also emphasizing the importance of cellular context in determining functional outcomes.

Beyond endothelial and cardiomyocyte compartments, Apelin has also been reported to modulate cardiac fibroblast activation. In pressure overload models, Apelin inhibits TGFβ-induced fibroblast activation and reduces α-smooth muscle actin expression and collagen production via suppression of sphingosine kinase 1 activity^109^. In myocardial infarction-induced heart failure, Apelin-13 attenuates fibrosis and improves systolic function, in part by suppressing Ang II-induced collagen type I and type III expression and TGFβ signaling through inhibition of PI3K-Akt signaling and oxidative stress pathways^110^. However, whether these effects reflect direct APLNR signaling in fibroblasts or indirect paracrine regulation remains to be fully resolved.

Taken together, current evidence most strongly supports a central role for endothelial APLNR in maintaining adaptive vascular support during cardiac remodeling under pressure overload. Reported cardiomyocyte and fibroblast effects likely reflect context-dependent, indirect, or developmentally restricted contributions. This distinction is critical for interpreting genetic and pharmacological studies targeting the Apelin–APLNR axis in heart failure and suggests that therapeutic strategies may benefit from prioritizing endothelial and paracrine signaling mechanisms.

## Apelin in endothelial inflammation and immune regulation

### Apelin in acute lung injury and acute respiratory distress syndrome

Multiple models of lipopolysaccharide (LPS)-induced acute lung injury (ALI) demonstrate strong anti-inflammatory and endothelial protective actions of Apelin-13. In a murine ALI model, Apelin-13 reduced structural lung damage, decreased the lung wet-to-dry ratio, and inhibits protein leakage into bronchoalveolar lavage fluid (BALF), accompanied by reduced fluid concentrations of TNFα, IL-6, and IL-1β^111^. Mechanistically, Apelin-13 inhibits nuclear factor (NF)-κB activation and prevents NLR family pyrin domain containing 3 (NLRP3) inflammasome assembly in lung tissue, which reduces IL-1β maturation and downstream inflammatory amplification^111^. A complementary study using mouse models and macrophage cultures showed that Apelin-13 attenuates LPS-induced ALI by suppressing NOX4-dependent reactive oxygen species production and limiting PFKFB3-driven glycolytic reprogramming in macrophages^112^. This reduction in metabolic activation decreases cytokine release and improves survival in ALI models^112^.

Clinically, serum Apelin-13 levels are elevated in patients with sepsis and sepsis-associated acute respiratory distress syndrome compared with healthy individuals, and longitudinal sampling suggests dynamic peptide regulation during critical illness^113^. Whether this rise reflects a protective response or a compensatory yet insufficient reaction remains unclear. Together, these findings position Apelin-13 as a regulator of acute pulmonary inflammation that functions by limiting NF-κB and NLRP3 activation, restraining reactive oxygen species-driven macrophage metabolism and preserving endothelial barrier integrity in mouse models of ALI.

### Apelin in post-injury pulmonary fibrosis and the endothelial–mesenchymal transition

Beyond acute injury, Apelin–APLNR signaling influences fibrotic remodeling in the lung. In a mouse model of LPS-induced pulmonary fibrosis, activation of the Apelin–APLNR axis reduces fibrosis severity and improves lung architecture^114^. In vitro studies show that Apelin-13 inhibits the LPS-induced endothelial–mesenchymal transition in lung microvascular ECs, whereas the Apelin–APLNR antagonist [Ala13]-Apelin-13 accelerates this transition^114^. These protective effects are closely tied to ACE2. Pharmacological inhibition of ACE2 abolishes the anti-fibrotic benefit of Apelin-13, indicating that Apelin–APLNR activation acts upstream of ACE2 to limit fibrotic remodeling^114^. LPS increases ACE2 ubiquitination, whereas Apelin-13 decreases ACE2 ubiquitination, suggesting that stabilization of ACE2 protein contributes to the anti-fibrotic role of Apelin-13 (ref. ^114^). Additional ALI-related fibrosis studies similarly report that Apelin-13 increases ACE2 expression, paralleling findings in cardiac hypertrophy models, and suppresses RAAS and NF-κB signaling to reduce endothelial–mesenchymal transition and collagen deposition^101,113,114^. Together, these data support a model in which Apelin–APLNR signaling protects the pulmonary microvasculature from chronic fibrotic remodeling by maintaining ACE2 expression and preserving endothelial function.

### Apelin–APLNR regulation of lung endothelial immune trafficking in EAE

EAE provides a model in which the lung acts as an intermediate priming site for autoreactive T cells before their entry into the central nervous system^115^. In this context, [Pyr^1^]Apelin-13 treatment in early EAE reduces immune cell accumulation and clustering in the lung, delays disease onset, and mitigates clinical severity^60^. These effects correlate with decreased intercellular adhesion molecule 1 (ICAM1) expression in pulmonary ECs and reduced inflammatory activation of the lung microvasculature^60^. In vitro, Apelin-13 reduces T cell adhesion to HUVECs in an APLNR-dependent manner, consistent with its ability to downregulate adhesion molecule expression and limit leukocyte–endothelium interactions^60^. These findings extend observations from ALI and lung fibrosis models by showing that Apelin can also modulate immune cell trafficking during autoimmune inflammation, not merely infection-induced injury. Spatial analyses further demonstrate that in EAE lungs, immune clusters preferentially localize next to *Aplnr*-positive ECs rather than *Apln*-positive cells^60^. In naive lungs, *Car4*^+^ aerocytes exhibit higher basal ICAM1 expression than Aplnr-positive capillaries. During EAE, however, ICAM1 is strongly induced in *Aplnr*-positive ECs as well as in *Car4*^+^ ECs^60^. This selective induction suggests that Aplnr-expressing microvessels function as inflammatory docking sites for leukocytes. The observation that Apelin-13 suppresses NLRP3 inflammasome activation^111^ is consistent with its ability to limit ICAM1 induction in Aplnr-positive ECs^60^, although the causal relationship between inflammasome inhibition and adhesion molecule expression remains unresolved.

### Endothelial junctions, Aplnr trafficking, and leukocyte transmigration

Apelin–APLNR signaling also regulates endothelial junctional organization. In HUVECs, GFP-tagged APLNR localizes to cell–cell junctions and disrupts VE-cadherin patterning, whereas [Pyr^1^]Apelin-13 treatment restores VE-cadherin localization while inducing Aplnr internalization^60^. Functionally, [Pyr^1^]Apelin-13 reduces T cell transendothelial migration in vitro, indicating that Apelin signaling can stabilize endothelial barriers during inflammatory activation^60^. In vivo, during EAE, Aplnr-positive ECs display lower junctional VE-cadherin expression than *Car4*^+^ aerocytes, but this difference is restored after [Pyr^1^]Apelin-13 treatment^60^. These findings suggest that receptor internalization induced by [Pyr^1^]Apelin-13 may alleviate APLNR-mediated junctional disruption and thereby reduce paracellular leukocyte passage.

Mechanistic insights into APLNR internalization come from heterologous systems. Structural and mutagenesis studies show that Phe255 and Trp259 in the receptor interact with the C-terminal phenylalanine of Apelin peptides to trigger receptor internalization without altering ligand binding or Gαi coupling^116^. Together with ligand-dependent β-arrestin recruitment and differential trafficking between Apelin-13 and Apelin-36 (ref. ^117^), these data indicate that APLNR internalization is tightly regulated and may contribute to desensitization or reprogramming of endothelial inflammatory responses.

### Apelin–APLNR regulation of endothelial junction integrity and leukocyte transmigration

Genetic studies highlight that APLNR functions extend beyond canonical Apelin-mediated signaling in vascular homeostasis. Although Apelin acts through APLNR to promote endothelial proliferation and sprouting in developmental and pathological angiogenesis models^65,118,119^, loss of Aplnr results in more severe vascular remodeling phenotypes than Apelin deficiency. By contrast, Apelin knockout mice display relatively mild baseline alterations and develop hypoxia-induced microvascular rarefaction and pulmonary muscularization primarily under stress conditions^77,120^. These differences likely reflect additional ligand-independent functions of APLNR, including mechanosensitive signaling, which are not captured by Apelin loss alone. In cardiomyocytes study, APLNR activation by mechanical stretch, even in the absence of Apelin, promotes hypertrophic signaling^72^. This observation raises the possibility that in mechanically dynamic tissues such as lung tissue, Aplnr may also mediate stretch-related inflammatory signaling, although this has not yet been demonstrated in pulmonary endothelium.

In EAE, *Aplnr* deletion reduces ICAM1 expression in pulmonary ECs, delays disease onset, and attenuates clinical severity, partially resembling the protective effects of [Pyr^1^]Apelin-13 treatment^60^. These findings are consistent with a model in which [Pyr¹]Apelin-13 may counteract potentially detrimental Aplnr activity by inducing receptor internalization^60,116^. However, whether this reflects suppression of genuine ligand-independent signaling or simply downregulation of ligand-driven pathways remains unclear and will require additional mechanistic investigation.

## Clinical translation and therapeutic development

Translational work targeting the Apelin–APLNR pathway has progressed from native peptide infusion to engineered peptide analogs and small-molecule agonists designed to address limitations observed in human disease. In patients with heart failure, cardiac *Apln* mRNA expression is elevated whereas circulating Apelin peptide levels are reduced, in part because ACE2 cleaves Apelin into shorter and less active fragments^42^. Studies of failing myocardium also report reduced APLNR expression compared with non-failing hearts^20^, indicating that both peptide instability and receptor downregulation constrain the activity of endogenous and infused Apelin in advanced heart failure. By contrast, recent single-cell transcriptomic analyses show that APLNR can be upregulated in specific EC states in human heart failure compared with donor hearts^121^, illustrating that receptor abundance varies according to disease context and cell identity. Early infusion studies in humans demonstrate that short-term APLNR activation is feasible. In healthy volunteers, [Pyr^1^]Apelin-13 increases the cardiac index and lowers peripheral vascular resistance, and patients with chronic heart failure display similar but attenuated responses^122^. These findings confirm that APLNR signaling remains accessible in heart failure, yet also highlight that disease-related changes in receptor abundance and downstream signaling limit the efficacy of native peptide infusion. Mechanistic studies further show that Apelin can increase ACE2 expression in failing hearts, suggesting a feedback loop in which Apelin–APLNR activation enhances a cardioprotective arm of the renin–angiotensin system even as ACE2 simultaneously degrades Apelin peptides^42,101^.

To overcome the very short half-life of native Apelin isoforms, metabolically stable peptide analogs have been developed. One example is the Apelin-17 analog LIT01-196, which incorporates a fluorocarbon chain at the N terminus and displays markedly increased stability and sustained in vivo activity^123^. In hypertensive deoxycorticosterone acetate salt rats, LIT01-196 normalizes blood pressure through an NO synthase-dependent mechanism without loss of efficacy^123^. More recent studies show that chronic LIT01-196 treatment after myocardial infarction improves left ventricular function, increases cardiac vascular density, and reduces adverse remodeling without lowering systemic blood pressure, indicating that APLNR signaling can enhance cardiac performance while minimizing hypotension risk^124^. These preclinical data suggest that stabilized Apelin analogs can reproduce and sustain key cardioprotective and vasodilatory effects of endogenous peptides.

Small-molecule agonists offer an oral alternative to peptide-based therapies. AMG 986 is a long-acting, orally available APLNR agonist that activates Gαi and β-arrestin signaling with sub-nanomolar potency^125,126^. In a first-in-human phase 1b study in healthy volunteers and in patients with heart failure, AMG 986 demonstrated an acceptable safety and pharmacokinetic profile across single and multiple dose regimens up to 650 mg per day, with nonlinear exposure increases and minimal accumulation^126,127^. Among patients with reduced ejection fraction, small numerical increases in left ventricular ejection fraction and stroke volume were observed, but these were inconsistent across imaging modalities and were not considered clinically meaningful during the short treatment period. Although short-term AMG 986 administration did not demonstrate meaningful pharmacodynamic benefit, its favorable safety profile supports further investigation of dose strategies, patient selection, and combination therapy approaches for APLNR agonists in heart failure^126^.

Human vascular disease studies demonstrate that altered APLNR expression is a key component of pathology and must be considered when designing receptor-directed therapies. In pulmonary veno-occlusive disease, expression of APLNR and the transcription factor ERG are markedly reduced in pulmonary venous ECs, and combined loss of *Erg* or *Aplnr* in mice produces venular occlusion and pulmonary hypertension that closely resemble the human condition^83^. Together with reduced myocardial APLNR expression in heart failure and context-dependent endothelial upregulation in single-cell RNA-sequencing datasets, these findings show that APLNR abundance is remodeled during disease progression. As a result, the effectiveness of Apelin or APLNR agonists is likely to depend on receptor availability within specific vascular beds and at particular stages of disease.

Beyond cardiovascular disease, circulating Apelin-13 is also being evaluated as a biomarker and potential modulator of immune-mediated pathology. Prior small cohort studies involving patients with multiple sclerosis reported inconsistent plasma Apelin-13 levels owing to variations in sex and disease state^128,129^. Park et al.^60^ recently quantified serum Apelin-13 in relapsing remitting multiple sclerosis and healthy controls and integrated these findings with EAE data. In female patients, serum Apelin-13 increased during relapse and remained moderately elevated during remission without statistical significance, approaching levels observed in healthy men, whereas disease status did not markedly affect Apelin-13 levels in male patients. These patterns support the potential use of serum Apelin-13 as a biomarker in female patients with multiple sclerosis, while also showing that baseline sex-related differences must be considered. Moreover, the protective effects of Apelin-13 treatment on endothelial activation and leukocyte trafficking in EAE suggest that both the magnitude and context of apelinergic signaling must be carefully evaluated when translating Apelin-based therapies targeting inflammatory disease.

Collectively, current translational studies demonstrate that Apelin–APLNR signaling can be safely targeted in humans and indicate that optimized peptide analogs and small-molecule agonists have promising preclinical benefits. At the same time, ACE2-mediated peptide degradation, a short peptide half-life, and disease-specific and cell-type-specific variation in APLNR expression limit the impact of native Apelin and simple infusion strategies. Development of future Apelin-based therapies will likely require metabolically stable agonists or small molecules combined with careful consideration of receptor expression pattern as well as sex-specific and disease-specific variations in circulating Apelin-13 and the broader renin–angiotensin–ACE2 axis.

## Conclusions and future perspectives

Together, current evidence positions the Apelin–APLNR axis as a predominantly endothelial signaling system that integrates vascular and immune responses in a context-dependent manner. Evidence for functional roles in other cell types, such as cardiomyocytes, remains less well defined, particularly given the low or context-dependent expression of APLNR, and warrants further investigation to better define potential cell-autonomous contributions. Studies across cardiovascular, pulmonary, and inflammatory models demonstrate that Apelin signaling regulates vascular tone, protects against ischemic and fibrotic injury, and modulates endothelial activation during immune responses. These findings support the view that Apelin acts as an endogenous vasoprotective and immunomodulatory factor, whose functional output is strongly shaped by disease context. Importantly, variation in Aplnr expression across organs, cell types, and pathological states, together with changes in circulating Apelin levels, indicates that the impact of this pathway cannot be generalized. Instead, interpretation of Apelin signaling requires careful consideration of receptor abundance and systemic peptide availability in each biological setting.

An important emerging concept is that APLNR signaling is not uniform but exhibits ligand-dependent and context-dependent bias. The balance between G protein-mediated signaling and β-arrestin recruitment critically determines downstream outcomes, including NO production, receptor desensitization, and long-term tissue remodeling. In this context, the development of biased APLNR agonists that preferentially enhance G protein signaling while minimizing β-arrestin engagement represents a promising strategy to improve therapeutic efficacy. Incorporating signaling bias into the conceptual framework of the apelinergic system will therefore be essential for translating mechanistic insights into clinically effective interventions.

Translational studies further indicate that although native Apelin peptides have beneficial biological effects, their therapeutic application is limited by rapid degradation, short half-lives, and disease-dependent variation in APLNR expression. Advances in peptide engineering and the development of small-molecule agonists have begun to address these limitations, with metabolically stable analogs and orally active compounds entering early clinical evaluation. Moving forward, successful therapeutic targeting of the apelinergic pathway will require integration of disease-specific receptor expression patterns, sex-dependent and state-dependent variation in circulating Apelin levels, and interactions with related systems such as the ACE2 axis. Optimizing pharmacokinetics, refining patient stratification, and aligning Apelin pathway modulation with existing therapies will be critical steps toward effective clinical translation and mechanistically informed vascular-targeted therapies.
